# Anti-inflammatory IL-10 administration rescues depression-associated learning and memory deficits in mice

**DOI:** 10.1186/s12974-020-01922-1

**Published:** 2020-08-22

**Authors:** Ryan J. Worthen, Susan S. Garzon Zighelboim, Camila S. Torres Jaramillo, Eleonore Beurel

**Affiliations:** 1 Department of Psychiatry and Behavioral Sciences, Miller School of Medicine, University of Miami, Gautier Building room 415, 1011 NW 15th Street, Miami, FL 33136 USA; 2 Department of Biochemistry and Molecular Biology, Miller School of Medicine, University of Miami, Gautier Building room, 4151011 NW 15th Street, Miami, FL 33136 USA

**Keywords:** Microglia, Learned helplessness, Interleukin-10, Learning and memory

## Abstract

**Background:**

Major depressive disorder is a widespread mood disorder. One of the most debilitating symptoms patients often experience is cognitive impairment. Recent findings suggest that inflammation is associated with depression and impaired cognition. Pro-inflammatory cytokines are elevated in the blood of depressed patients and impair learning and memory processes, suggesting that an anti-inflammatory approach might be beneficial for both depression and cognition.

**Methods:**

We subjected mice to the learned helplessness paradigm and evaluated novel object recognition and spatial memory. Mice were treated with IL-10 intranasally or/and microglia cells were depleted using PLX5622. Statistical differences were tested using ANOVA or *t* tests.

**Results:**

We first established a mouse model of depression in which learning and memory are impaired. We found that learned helplessness (LH) impairs novel object recognition (NOR) and spatial working memory. LH mice also exhibit reduced hippocampal dendritic spine density and increased microglial activation compared to non-shocked (NS) mice or mice that were subjected to the learned helpless paradigm but did not exhibit learned helplessness (non-learned helpless or NLH). These effects are mediated by microglia, as treatment with PLX5622, which depletes microglia, restores learning and memory and hippocampal dendritic spine density in LH mice. However, PLX5622 also impairs learning and memory and reduces hippocampal dendritic spine density in NLH mice, suggesting that microglia in NLH mice produce molecules that promote learning and memory. We found that microglial interleukin (IL)-10 levels are reduced in LH mice, and IL-10 administration is sufficient to restore NOR, spatial working memory, and hippocampal dendritic spine density in LH mice, and in NLH mice treated with PLX5622 consistent with a pro-cognitive role for IL-10.

**Conclusions:**

Altogether these data demonstrate the critical role of IL-10 in promoting learning and memory after learned helplessness.

## Introduction

Depression is a prevalent and debilitating disease that affects ~ 17% of the US population [27] and represents an important economic burden [9]. Depressed patients often experience attention, concentration, perception, executive function, and processing speed deficits, in addition to depression symptoms, that hamper everyday functions [50, 64]. Beck’s cognitive model of depression developed over 50 years ago postulated that the development and maintenance of depression relies on biased acquisition and processing of information [5], suggesting that cognition bias is at the heart of depressive pathogenesis. The functional and neurobiological architecture of Beck’s model has been identified, involving in part the limbic system [18]. However, little is known about the mechanisms underlying cognitive impairment in depression [21].

Inflammation and cytokines are of particular interest because they are involved in both cognitive impairment and the development of depression. Thus, on one hand, pro-inflammatory cytokines impair hippocampal-dependent memory [for reviews: [36, 62]], even though their contribution to homeostatic cognition remains unclear. On the other hand, pro-inflammatory cytokines also promote depressive-like behaviors in mice [16, 46]. Furthermore, psychological stress induces pro-inflammatory cytokine production [15, 46]. In contrast, anti-inflammatory cytokines, such as interleukin (IL)-10, alleviate deleterious effects of cytokines on memory and plasticity [35, 48], blocking for example the detrimental effects of lipopolysaccharide (LPS) or IL-1β on long-term potentiation (LTP) [26, 35, 42], and rescuing learning and memory deficits in inflammation-dependent models of Alzheimer’s disease [25, 29, 30]. Furthermore, a higher circulating IL-10 level has been associated with lower levels of self-reported stress as well as higher measures of cognitive function [24]. Consistent with this, IL-10 levels are reduced in major depressive disorder (MDD) patients [10, 17] although not all [55], and IL-10 attenuates LPS-induced depressive-like behaviors in rats [11], whereas antidepressants increase IL-10 levels [31, 33, 55]. Taken together, IL-10 may have beneficial actions both within cognition and depression.

The production of cytokines after stress has been attributed mainly to microglia in the brain. Microglial activation is induced by stress in various brain areas and has been associated with depressive-like behaviors in rodents [review [60]]. During homeostasis, microglial cells play an important role in neuroplasticity because of their crucial role in clearing apoptotic newborn neurons or dendritic spines through phagocytosis [53, 61] and in secreting factors that are required for the proliferation, differentiation, and migration of newborn neurons [1, 4, 14, 58], promoting neurogenesis, which has been reported to be impaired in rodent models of depression [40]. Furthermore, environmental enrichment, which confers resilience to stress and provides antidepressant effects, induces a mild microglial activation, similarly to LPS, macrophage colony-stimulating factor (M-CSF), or granulocyte-macrophage colony-stimulating factor (GM-CSF), suggesting that the degree of microglial activation might be critical to determine the outcomes on functionality of neuroplastic circuits. Finally, minocycline administration, commonly used to block microglial activation, blocks depressive-like behavior in mice and ameliorates depressive symptoms in humans [32, 47], and this was accompanied by a decrease in inflammatory cytokine production.

Here, we developed a new mouse model that exhibits learning and memory impairments after induction of learned helplessness, which was associated with activated microglia and reduced dendritic spine density. These impairments were reversed by intranasal administration of IL-10, demonstrating the importance of IL-10 in resilience to stress.

## Material and methods

### Mice and drug administration

These studies were conducted using male adult (8–12 weeks old) C57BL/6 wild-type, GSK3α/β^21A/21A/9A/9A^ knock-in, and *Fmr1*^−/−^ mice. Female mice were used in Suppl Fig 5. When indicated, mice were treated intranasally (i.n.) with phosphate-buffered saline (PBS) or murine recombinant IL-10 (5 μg/mouse, Peprotech) 24 h and 1 h prior to behavioral testing. When indicated, mice received 10 μg/mouse siRNA targeting STAT3 (69367, Ambion) or scrambled siRNA control (Silencer Negative Control #5 siRNA, Ambion) 24 h prior to subjecting mice to behavioral testing and every 24 h for the subsequent 3 days until the end of the experiment. Depletion of microglia was achieved using PLX5622 (1200 ppm, Plexxikon) present in the diet (AIN-76A). Mice were fed ad libitum for a minimum of 3 days before each experiment, and the diet was maintained for the duration of the experiment. Mice were bred in the University of Miami animal facility. Mice were housed in standard cages in light- and temperature-controlled rooms and were treated in accordance with NIH and the University of Miami Institutional Animal Care and Use Committee regulations.

### Behaviors

#### Learned helplessness

The learned helplessness paradigm was used to induce depressive-like behavior in mice as described previously [7]. Mice were placed in one of two chambers (Med Associates, St. Albans, VT, USA) separated by a closed gate. Mice received 180 inescapable foot-shocks (IES) of 0.3 mA intensity and an average duration of 6–10 s with random and unpredictable inter-shock intervals ranging from 5 to 45 s. On the following day, mice were reintroduced to the same apparatus. Once the 0.3 mA foot-shocks began, the gate separating both chambers opened, giving each mouse a maximum of 24 s to escape the shock by shuttling to the opposite chamber. The shock was terminated when the mouse crossed the gate. Mice were tested in 30 escapable foot-shock (ES) trials, and the mice that failed to escape more than 15 out of 30 trials were considered learned helpless.

#### Learning and memory

Mice were acclimated to the behavioral testing room for at least 30 min prior to testing in the presence of a white noise generator (55 dB) and were assessed twice for each behavior, before and after treatment. All sessions were video-recorded, and all videos were analyzed blind to the treatment.

##### Novel object recognition (NOR)

A baseline measurement of NOR was conducted 1 day after the last escapable foot-shock trials. This task takes advantage of a mouse’s natural inclination to preferentially explore novel objects it encounters compared to previously encountered, familiar objects. The amount of time mice spent exploring a novel object compared to a familiar object provides an indication of recognition memory [3]. Two identical copies of object 1 (125-mL glass bottles with black cap, 4–6 cm in diameter × 10 cm in height) were placed in a Plexiglas arena (50-cm long × 20-cm wide × 25-cm tall) equidistant from the walls of the container. Mice were individually placed within the arena and were allowed to explore the objects during a 5-min habituation phase. Following this, mice were removed from the arena and placed in an opaque holding container for 5 min. Mice were reintroduced to the same arena and presented with one copy of object 1 as well as a novel, never previously encountered object 2 (translucent red Plexiglas bottle, 8 cm in diameter × 13 cm in height). The total time each mouse spent exploring object 1 (familiar, F) compared to object 2 (novel, N) was recorded (exploration was defined as touching the object with the nose or forelimbs, sniffing the object, or closely approaching the object in a forward, attention-directed lunge) as we previously reported [45]. Both the percent of time spent with each object and the discrimination ratio [(time spent with N-time spent with F)/(time spent with N and F)] was reported. After IL-10 treatment, NOR was re-assessed with a new set of objects (object 1: blue bottle cap with metal nozzle, 6 cm in diameter × 4 cm in height; object 2: black rubber bottle stopper, 4 cm in diameter × 7 cm in height) in the same cohort of mice.

##### Two-trial Y-maze

A two-trial Y-maze was used to measure spatial working memory [19]. The Y-maze apparatus (Stoelting Co.) (lane width 5 cm × arm length 35 cm × arm height 10 cm) was arranged on an elevated platform with one arm facing outward toward the experimenter (start arm, S) and two distal arms facing inward toward the back wall of the room. Extra-maze visual cues of varying shapes and sizes were placed on the walls of the room ranging from 0.5 to 2 m distance from the maze itself. During a preliminary acquisition phase, the left or the right distal arm was obstructed by placing a barrier block at the entrance of the arm. Mice were individually placed into the end of the start arm and allowed to explore the open areas of the maze for 5 min. Following this, mice were removed from the maze and returned to their home cage during a 30-min inter-trial interval (ITI). During a 2-min retrieval trial, mice were returned to the start arm and allowed to freely explore all arms, including the previously obstructed, novel arm (N). Measurement of exploratory behavior began when a mouse had left the start arm, and the total time each mouse spent exploring the novel arm compared to the familiar arm was calculated. An arm entry was defined as placing all four paws within the arm, and a mouse was considered to have exited an arm when all four paws were located outside of the arm. Periods in which a mouse engaged in self-grooming or remained stationary were excluded from the final calculation. After IL-10 treatment, mice were reassessed in the Y-maze in a different room with a distinct set of extra-maze visual cues. Mice were assessed using a 1-min ITI between acquisition and retrieval trials to confirm preference for novelty, sufficient visual acuity to recognize extra-maze visual cues, and to control for potential anxiety-like motivational disturbances that may have influenced exploratory behavior.

### Flow cytometry

After behavioral testing, mice were anesthetized with isoflurane and transcardially perfused with PBS, and hippocampi were recovered. The hippocampi were passed through a 70-μm cell strainer (BD Bioscience), and the cell suspension was mixed (vol/vol) to obtain a 30% Percoll/R1 medium that was overlaid on 70% Percoll/R1 medium in a centrifuge tube, and centrifuged at 2000 rpm for 20 min without using the brake. The cells at the interface of the 30/70% Percoll gradient were recovered, washed one time, resuspended in R10 media, aliquoted into a 96-well plate, and incubated for 4 h with the Protein Transport Inhibitor Cocktail (eBioscience) at the recommended concentrations. Standard intracellular cytokine staining was carried out as described [23] using the Staining Intracellular Antigens for Flow Cytometry Protocol (eBioscience). Cells were first stained extracellularly with FITC-conjugated anti-CD45 (to identify microglial cells), and then were stained intracellularly with PcBlue-conjugated anti-IL-10. Samples were acquired on a Celesta (BD) flow cytometer, and data were analyzed with the FlowJo software (Tree Star, Inc.).

### Immunofluorescence

After the learned helplessness paradigm, mice were anesthetized with isoflurane and transcardially perfused with 0.9% NaCl and 4% paraformaldehyde (PFA; #P614, Sigma-Aldrich). Brains were extracted and placed in 4% PFA overnight at 4 °C and stored in 30% sucrose, 0.02% sodium azide in phosphate buffer (pH 7.4) until sectioning. Brains were rapidly embedded in optimal cutting temperature compound (O.C.T., Tissue-Tek) in a mold (Sigma-Aldrich). Frozen sagittal brain sections of 20-μm thickness were prepared using a cryostat (Leica). Free-floating sections were washed in PBS (pH 7.5), incubated in PBS containing 0.1% Triton-X for 10 min, and blocked in PBS containing 5% normal donkey serum for 1.5 h. Sections were then incubated overnight at 4 °C in primary antibody of anti-AIF-1/Iba1 (1:500, #NB100-1028, Novus Biologicals) or anti-STAT3 (1:1000, #12640, Cell Signaling Technology). Sections were washed in PBS, incubated with donkey anti-goat Alexa 594-conjugated secondary antibody (1:1000; Life Technologies) or chicken anti-rabbit Alexa 488-conjugated secondary antibody (1:1000; A-21441 Life Technologies) overnight at 4 °C. Following washes in PBS, tissue was mounted on a glass microscope slide and cover-slipped using VectaShield mounting medium with DAPI (Vector Laboratories). Hippocampal images were acquired using an Olympus FLUOVIEW FV1000 confocal laser-scanning microscope with 40X magnification housed at the Microscopy Core Facility, or a VS120 Olympus slide scanner with 40X magnification housed at the Analytical Imaging Shared Resource, at the University of Miami. Individual confocal stacks were merged to form composite images containing the entire hippocampus, which were then analyzed blinded to treatment using standardized parameters with the ImageJ software. For each mouse, quantification of Iba1-positive microglia was performed by counting the total number of positive cells within a sampling area comprised of 5 separate 200 μm x 200 μm square ROIs (combined total area of 0.2 mm^2^) superimposed and distributed randomly over the dentate gyrus. Sampling areas within 2–4 hippocampal slices per animal were analyzed, and the average number of Iba1-positive microglia within the dentate gyrus was determined for each mouse. Furthermore, microglial sphericity was evaluated in the dentate gyrus using Image J. Ten well-resolved microglial cells were analyzed per image in 2–4 hippocampal slices per animal. The perimeter of the cell body was traced, and microglial cross-sectional area (μm^2^) was calculated as a measure of soma size. Additionally, the length (μm) of the longest cell process emanating from the cell body was measured to indicate the scale of cell process branching.

### Golgi staining

After the learned helplessness paradigm, mice were sacrificed, and the brains were recovered to undergo Golgi staining according to the manufacturer’s instructions (FD Neurotechnologies, Inc.). Once fixed, the brains were flash-frozen and 120-μm coronal sections were prepared and mounted onto gelatin-coated glass slides. After allowing the tissue to dry and adhere to the slides for 1 week at 35 °C, samples were dehydrated with sequentially increasing percentage ethanol/ddH_2_O solutions and xylene, and finally slides were cover-slipped using Eukitt (#NC9068612, Thermo Fisher Scientific) hardening mounting medium. Hippocampal images were acquired at 100X magnification with the EvosXL Core light microscope (Life Technologies). At Bregma − 2.30 mm, apical dendrites, 20 μm from the neuronal cell body, were imaged within the CA1, CA3, and dentate gyrus anatomical regions of the dorsal hippocampus. Images were analyzed blinded to treatment, and dendritic spine density was quantified using the Neurolucida and Neuroexplorer software, available in the Microscopy Core Facility at the University of Miami, or ImageJ. The average number of dendritic spines over approximately 80 μm of total apical dendrite length (not necessarily from the same neuron) per hippocampal region was determined for each mouse.

### Immunoblot analysis

Mice were sacrificed by decapitation 1 h after i.n. administration of either vehicle (PBS) or IL-10 (5 μg/mouse). Brains were extracted and the prefrontal cortex, hippocampus, and cerebellum were dissected. Samples were homogenized in lysis buffer containing 20 mM Tris-HCl, pH 7.4, 150 mM NaCl, 1 mM EDTA, 1 mM EGTA, 1% Triton-100, 10 μg/mL leupeptin, 10 μg/mL aprotinin, 5 μg/mL pepstatin, 1 mM phenylmethanesulfonylfluoride fluoride, 1 mM sodium orthovanadate, 50 mM sodium fluoride, and 100 nM okadaic acid. Tissue homogenates from the 3 brain regions were centrifuged at 14,000 rpm for 10 min at 4 °C to remove cellular debris, and the supernatant was recovered. Protein concentration was evaluated using the Bradford method. Thirty micrograms of protein was loaded onto a 10% SDS polyacrylamide gel and transferred into a nitrocellulose membrane. The membrane was blocked and incubated with primary anti-phospho-Tyr^705^-STAT3 or anti-STAT3 antibodies overnight at 4 °C, followed by an overnight incubation at 4 °C with a secondary antibody conjugated to horseradish peroxidase (HRP). Signals were visualized using chemiluminescence with an Amersham Imager600 and quantified using the iQTL software (GE Healthcare). Blots were reblotted for β-actin to ensure proper loading.

### Statistical analysis

Data were represented as mean ± SEM. Results were analyzed using Student’s *t* test or one- or two-way analysis of variance (ANOVA) with a Bonferroni or Fisher’s LSD *post hoc* test using the Prism software as indicated; *p* values of less than 0.05 were considered statistically significant (suppl table 1).

## Results

### Microglia are activated after learned helplessness

**Fig. 1:**
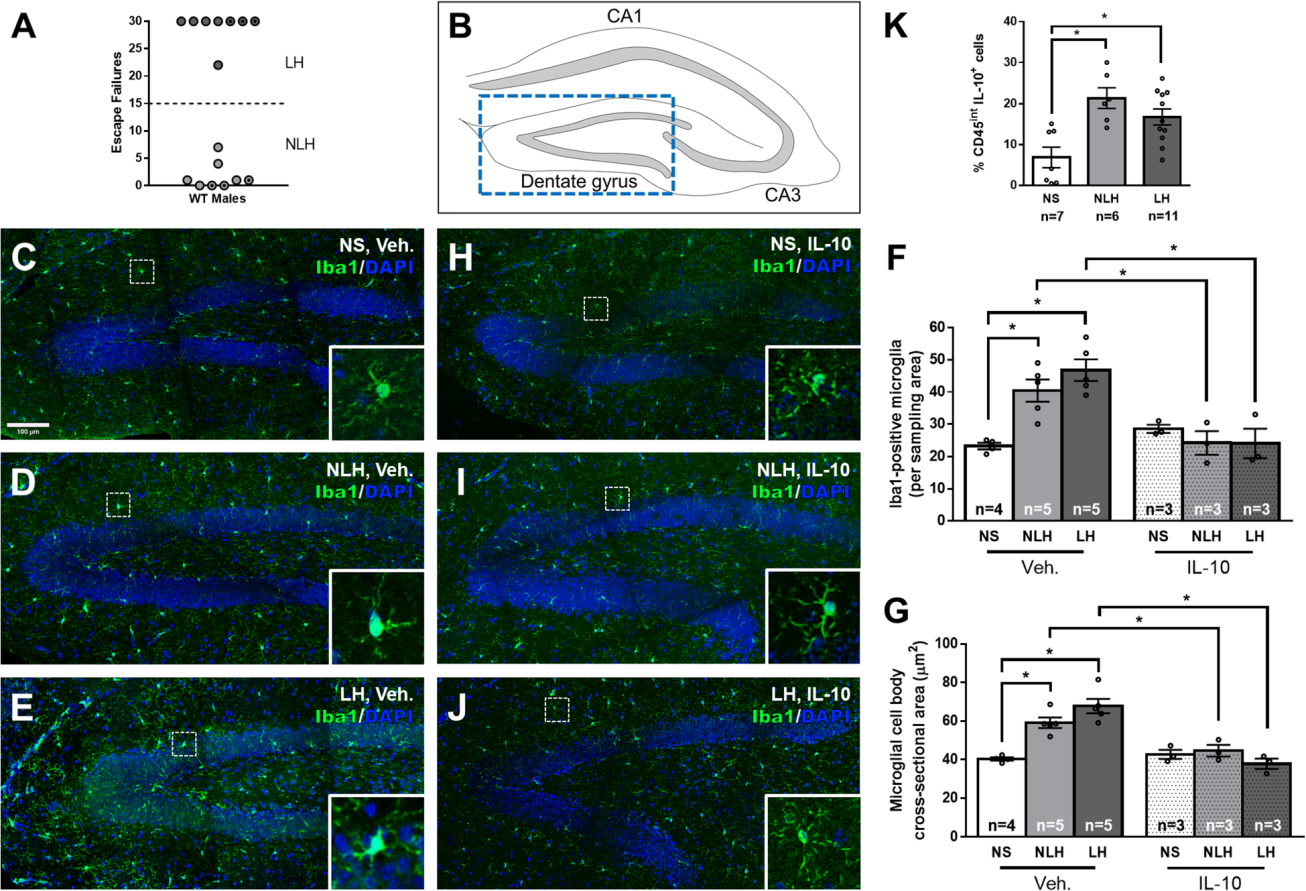
Microglial activation increased in stressed mice and was alleviated by IL-10 treatment. Male wild-type mice were subjected or not (NS) to the learned helplessness paradigm and separated into 2 groups: non-learned helpless (NLH) and learned helpless (LH) mice, according to their number of failures out of 30 escapable shock trials (**a**) and were treated or not with vehicle (Veh.) or IL-10 (5 μg/mouse) 24 h and 1 h prior to sacrifice. Mice were perfused and brains were collected for immunostaining. **b** Diagram of the dorsal mouse hippocampus in cross-section highlighting three major anatomical regions of the perforant pathway: CA1, CA3, and the dentate gyrus (DG). Representative images of Iba1^+^ microglial cells in the dentate gyrus of vehicle-treated: non-shocked control (NS) (**c**), NLH (**d**), and LH (**e**) mice, and IL-10-treated: NS (**h**), NLH (**i**), and LH (**j**) mice. Enlarged images of representative microglial cells (white dashed outline) exhibiting the predominant morphology are included in each panel. **f** Number of Iba1^+^ microglial cells per 200,000 μm^2^ sampling area within the dentate gyrus for each mouse. Each dot represents a mouse. Two-way ANOVA, F(2,21)_interaction_ = 10.60, F(1,21) _treatment_ = 17.44, F(2,21)_condition_ = 5.865, Bonferroni post hoc test, *p < 0.05, bars represent means ± SEM, *n* = 3-5 mice/group. **g** Microglial morphology (sphericity) was measured as the microglial cell body cross-sectional area within the dentate gyrus for each mouse. Each dot represents a mouse. Two-way ANOVA F(2,17)_interaction_ = 13.99, F(1,17)_treatment_ = 32.02, F(2,17)_condition_ = 8.281, Bonferroni post hoc test, *p < 0.05, bars represent means ± SEM, *n* = 3-5 mice/group. **k** Percentage of CD45^int^IL-10^+^ microglial cells in the hippocampus of NS, NLH, and LH mice, measured by flow cytometry. Each dot represents a mouse. One-way ANOVA, F(2,21) = 8.895, Bonferroni post hoc test, *p < 0.05, bars represent mean ± SEM, *n* = 6–11 mice/group

Because stress induces microglial activation [61], we first tested if microglial cells were activated after the induction of learned helplessness. Exposure to the learned helplessness protocol produced two groups of mice, those that exhibited learned helplessness (mice failed to escape > 15/30 trials, LH mice), and mice that did not exhibit learned helplessness (mice failed to escape < 15/30 trials, NLH mice) (Fig. 1 a). These two groups of mice were compared to non-shocked (NS) mice following Iba1 immunostaining to detect activated microglial cells. The number of Iba1^+^ microglial cells in the dentate gyrus of the hippocampus (Fig. 1 b) increased ~ 1.7 to 2-fold 48 h after the learned helplessness protocol in both NLH and LH mice compared to NS mice (Fig. 1 c–f). This indicates that foot-shocks are sufficient to increase the number of Iba1^+^ microglial cells. Similar increases in the number of Iba1^+^ microglial cells were observed in the CA1 and CA3 regions of the hippocampus (data not shown) suggesting that microglia were activated by foot-shocks throughout the hippocampus. In addition, Iba1^+^ microglial cells in the dentate gyrus displayed an amoeboid morphology as exemplified by a bigger soma area, in mice subjected to the learned helplessness paradigm, whether they exhibited learned helplessness or not, compared to non-shocked mice that had microglia that exhibited a more quiescent, ramified phenotype (Fig. 1 c–e, g). These results indicate that stress caused by foot-shocks activates hippocampal microglial cells, which may contribute to behavioral responses to stress.

### IL-10 production increased in stressed mice

We also tested if activated microglia produce anti-inflammatory IL-10 after learned helplessness, using flow cytometry to identify IL-10^+^ microglial cells (Suppl Fig 1A). We found that hippocampal IL-10^+^CD45^int^ cells were significantly 3- and 2.4-fold increased in NLH and LH mice respectively compared to NS mice (Fig. 1 k). The proportion of microglial cells expressing IL-10 was reduced by 20% in the LH mice compared to the NLH mice. This is consistent with previous reports of lower brain IL-10 in mice that display depressive-like behavior and demonstrate that deficient microglial IL-10 contributes to this deficit [39].

### IL-10 prevents foot-shock-induced microglial activation

To ensure microglial activation was associated with a microglial inflammatory profile, we tested if administration of the anti-inflammatory cytokine IL-10 was capable of reversing the activated phenotype of microglial cells in the dentate gyrus. We administered IL-10 intranasally and found IL-10-downstream signaling, STAT3 Tyr705-phosphorylation increased 1 h after IL-10 treatment in several brain regions, including the hippocampus (Suppl Fig 1B), indicating that intranasal administration of IL-10 lead to STAT3 activation in the brain. Intranasal IL-10 treatment did not affect Iba1 staining in NS mice but reduced microgliosis by 40% and 48% in the dentate gyrus of NLH and LH mice, respectively, compared to vehicle-treated NLH and LH mice (Fig. 1 f, h–j), reaching similar levels of Iba1^+^ cells detected in NS mice. These results indicate that stress promotes microglial activation that can be alleviated with IL-10 treatment.

### Dendritic spine density was reduced in learned helpless mice, and IL-10 administration promotes dendritic spine density

**Fig. 2:**
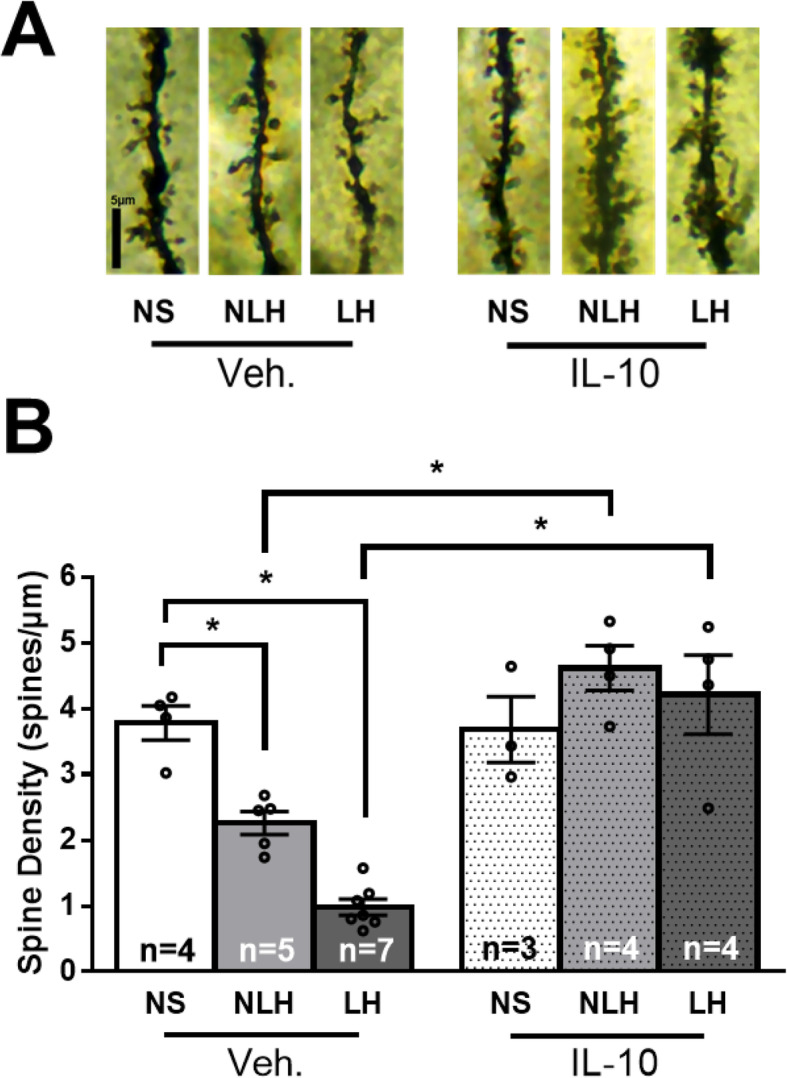
The decrease of hippocampal DG dendritic spine density in stressed mice was abolished by IL-10 treatment. Male wild-type mice were subjected or not (NS) to the learned helplessness paradigm and separated into 2 groups: non-learned helpless (NLH) and learned helpless (LH) mice, according to their number of failures out of 30 escapable shock trials and were treated or not with vehicle (Veh.) or IL-10 (5 μg/mouse) 24 h and 1 h prior to sacrifice. **a** Representative images of dendritic spines on apical dendrites radiating from granule cells within the dentate gyrus (DG). **b** Dendritic spine density was calculated as an average per animal (# spines/μm) over 80 μm of total analyzed apical dendrite length (20 μm from the cell body) extending from granule cells within the molecular layer of the DG region of the dorsal hippocampus. Each dot represents a mouse. Two-way ANOVA F(2,33)_interaction_ = 14.33, F(1,33)_treatment_ = 26.32, F(2,33)_condition_ = 2.948, Bonferroni post hoc test, *p < 0.05, bars represent mean ± SEM, *n* = 5–8 mice/group

Because microglia are important for synaptic pruning [43], we examined if dendritic spine density was affected by the induction of learned helplessness, which activated microglial cells. Consistent with the findings that stress reduces dendritic spine density in the hippocampus [52], we found that dendritic spine density was decreased by 1.8-fold in the dentate gyrus of NLH mice and was decreased significantly more, 3.2-fold, in the dentate gyrus of LH mice (Fig. 2). Similar differences were evident in the CA1 and CA3 regions of the hippocampus (Suppl Fig 2). These findings confirm that foot-shock stress is sufficient to reduce hippocampal dendritic spine density, and further demonstrate that greater hippocampal dendritic spine density reductions are evident in mice that develop learned helplessness.

Intranasal administration of IL-10 increased dendritic spine density by 2.0- and 4.3-fold in the dentate gyrus of NLH and LH mice, respectively, compared to vehicle administration. Furthermore, after IL-10 treatment, the dendritic spine density was increased 1.3- and 1.1-fold in the dentate gyrus of NLH and LH mice compared to NS mice treated with IL-10 (Fig. 2), suggesting that IL-10 not only enhanced dendritic spine density in the hippocampus but it enhanced it above basal levels. Altogether, this data suggests that since hippocampal dendritic spine density was greatly reduced in LH mice, cognitive performance after learned helplessness might also be impaired, and that IL-10 administration might reverse such impairments.

### Learned helpless mice exhibited microglia-dependent impaired learning and memory

**Fig. 3:**
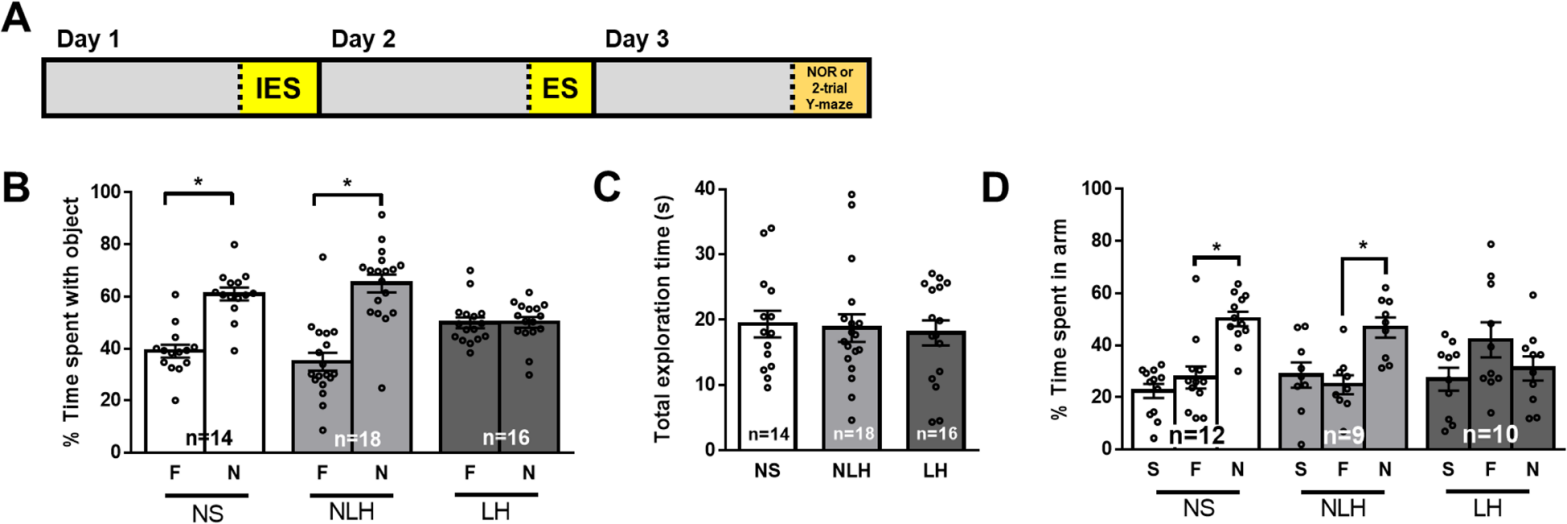
Learned helpless mice exhibited impaired novel object recognition and spatial memory. Male wild-type mice were subjected or not (NS) to the learned helplessness paradigm and separated into 2 groups: non-learned helpless (NLH) and learned helpless (LH) mice, according to their number of failures out of 30 escapable shock trials, and learning and memory were assessed the next day. **a** Timeline of the experiment. The percentage of a mouse’s total object exploration time spent exploring the familiar (F) and novel (N) objects (**b**) and the total time (s) mice spent engaged in exploration of both the familiar and novel objects combined (**c**) in the novel object recognition test were reported. Each dot represents a mouse. Two-way ANOVA, F(2,90)_interaction_ = 15.53, F(1,90)_treatment_ = 56.16, F(2,90)_condition_ = 4.238 × 10^−14^, Bonferroni post hoc test, *p < 0.05, compared to % time spent with familiar object, bars represent means ± SEM, *n* = 14–18 mice/group. **d** The percentage of a mouse’s total maze exploration time spent exploring the start (S), familiar (F), and novel (N) arms in the two-trial Y-maze test was measured in a different cohort of mice than **b**, **c**. Each dot represents a mouse. Two-way ANOVA, F(4,84)_interaction_ = 5.373, F(2,84)_treatment_ = 11.68, F(2,84)_condition_ = 7.654 × 10^−14^, Fisher’s least significant difference test, *p < 0.05, compared to % time spent exploring familiar arm, bars represent means ± SEM, *n* = 9–12 mice/group

We tested if exposure to the learned helplessness protocol affected learning and memory in two tasks, novel object recognition and spatial working memory (Fig. 3 a). We were particularly interested in determining if cognitive impairments were linked to the development of learned helplessness or were merely caused by the stress of the protocol. Mice were subjected to the learned helplessness paradigm, and 24 h after the last escapable foot-shocks, novel object recognition or spatial working memory were tested in two different cohorts of mice. LH mice exhibited impaired novel object recognition, as they spent similar amounts of time exploring the familiar and the novel objects and they exhibited a reduced discrimination index (Fig. 3 b and Suppl Fig 3A). In contrast, NLH mice exhibited preference for the novel object equivalent to NS mice, demonstrating intact novel object recognition. All mice spent similar amounts of time exploring the objects (Fig. 3 c). These findings indicate that the stress of the LH protocol did not impair novel object recognition, but impairment was evident in mice that displayed learned helplessness.

Similar results were obtained using the two-trial Y-maze, LH mice spent similar amounts of time in the familiar and novel arms, indicative of cognitive impairment. In contrast, both NLH and NS mice spent more time in the novel arm than the familiar arm (Fig. 3 d). Yet, LH mice preferred the novel arm in the 1-min ITI protocol, suggesting that the impairment seen in LH mice was not the result of anxiety or inability to recognize the cues (Suppl Fig 3B-G). As with the novel object recognition task, this indicates that the development of learned helplessness and depressive-like behavior, but not exposure to the foot-shock protocol, caused learning and memory impairment. Thus, LH mice exhibited impaired novel object recognition and impaired spatial memory that were not impaired in NLH mice exposed to the learned helplessness protocol.

**Fig. 4:**
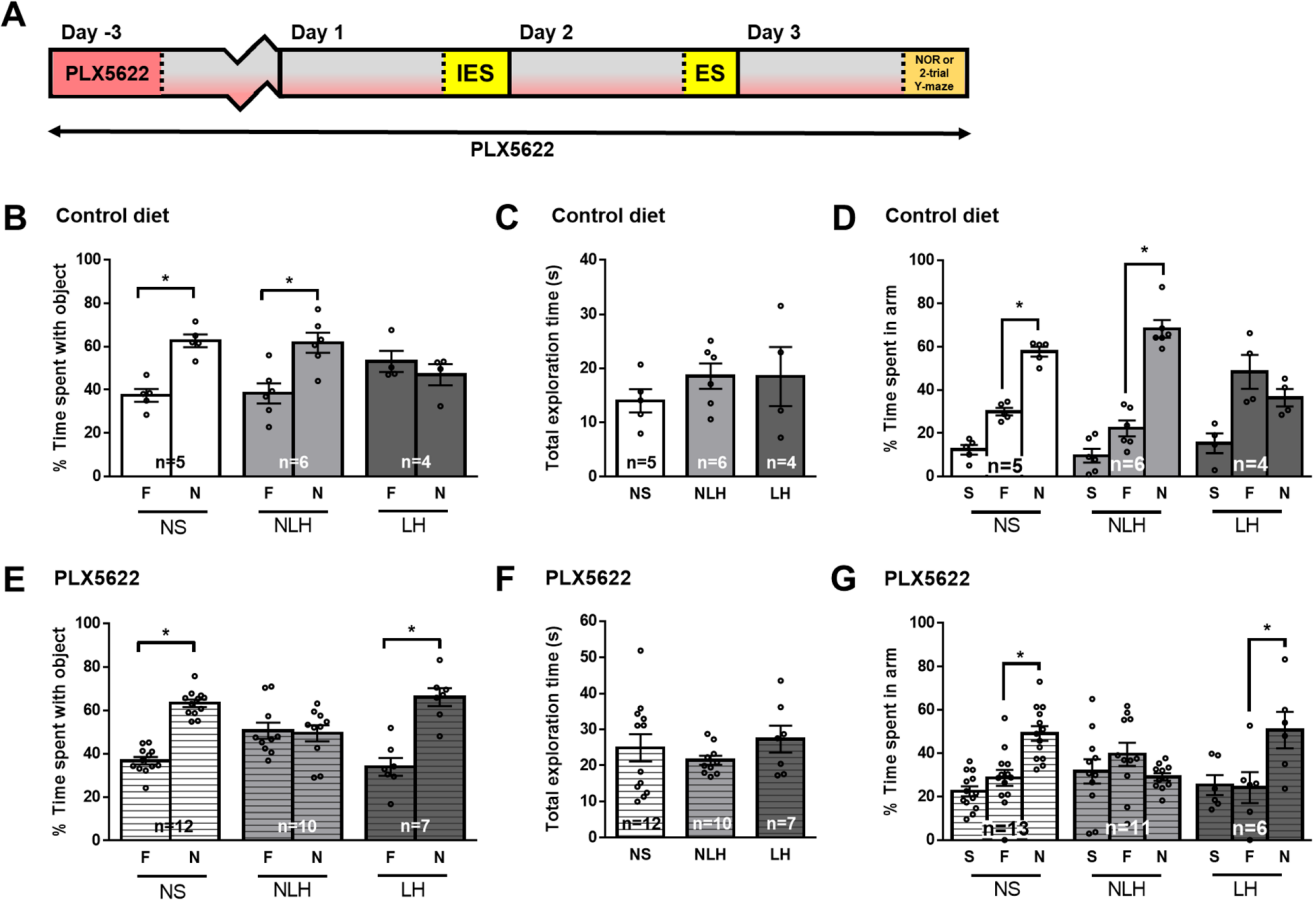
Depletion of microglial cells is sufficient to rescue the learning and memory impairments of LH mice. Male mice were fed with the AIN-76A diet (control diet) (**b**–**d**) or AIN-76A diet supplemented with PLX5622 (**e**–**g**) for 3 days before the induction of the learned helplessness paradigm and were maintained on the diet for the duration of the experiment. Three days after initiating the diet, mice were subjected or not (NS) to the learned helplessness paradigm and separated into 2 groups: non-learned helpless (NLH) and learned helpless (LH) mice, according to their number of failures out of 30 escapable shock trials, and the next day, learning and memory was assessed. **a** Timeline of the experiment. The percentage of a mouse’s total object exploration time spent exploring the familiar (F) and novel (N) objects (**b**, **e**) and the total time (s) mice spent engaged in exploration of both the familiar and novel objects combined (**c**, **f**) in the novel object recognition test were reported. Each dot represents a mouse. Two-way ANOVA F(2,24)_interaction_ = 7.334, F(1,24)_treatment_ = 15.83, F(2,24)_condition_ = 5.380 × 10^−14^, Bonferroni post hoc test, *p < 0.05, compared to % time spent with familiar object, bars represent means ± SEM, *n* = 4–12 mice/group. **d**, **g** The percentage of a mouse’s total maze exploration time spent exploring the start (S), familiar (F), and novel (N) arms in the two-trial Y-maze test was measured in a different cohort of mice than **b** and **e**. Each dot represents a mouse. Two-way ANOVA, F(4,81)_interaction_ = 5.807, F(2,81)_treatment_ = 9.980, F(2,81)_condition_ = 0.0, Fisher’s least significant difference test, *p < 0.05, bars represent means ± SEM, *n* = 4–13 mice/group

To test if microglial cells were involved in the cognitive impairments of LH mice, mice were treated for 3 days with PLX5622, which eliminated ~ 90% of microglial cells [Suppl Fig 4A-C, [2], prior to behaviors (Fig. 4 a), and similar elimination of microglia cells was found after 6 days of PLX5622 (data not shown). LH mice receiving the control diet (AIN-76A diet) without PLX5622 exhibited similar novel object recognition or spatial memory impairments as mice receiving the regular diet used by the animal facility at the University of Miami (Fig. 4 b–d). Elimination of microglial cells with PLX5622 treatment did not affect novel object recognition or spatial memory of NS mice (Fig. 4 e–g, Suppl Fig 3A). However, PLX5622 treatment restored novel object recognition and spatial memory of LH mice (Fig. 4 e–g, Suppl Fig 3A), indicating that microglial cells contributed to these cognitive deficits associated with LH. PLX5622 also reduced the proportion of mice exhibiting learned helplessness (Suppl Fig 4D). In line with evidence that microglia have important roles in healthy brain function as well as detrimental actions in diseased brains, microglial depletion with PLX5622 treatment impaired novel object recognition and spatial memory of NLH mice (Fig. 4 e–g, Suppl Fig 3A), which were not impaired in the absence of PLX5622 (Fig. 4 b–d, Suppl Fig 3A). These findings indicate that microglial cells in the LH mice contribute to impaired learning and memory, whereas microglial cells contribute to a healthy stress-response in supporting cognition in the stressed but NLH mice.

### IL-10 reversed LH-dependent recognition and spatial memory impairments

**Fig. 5:**
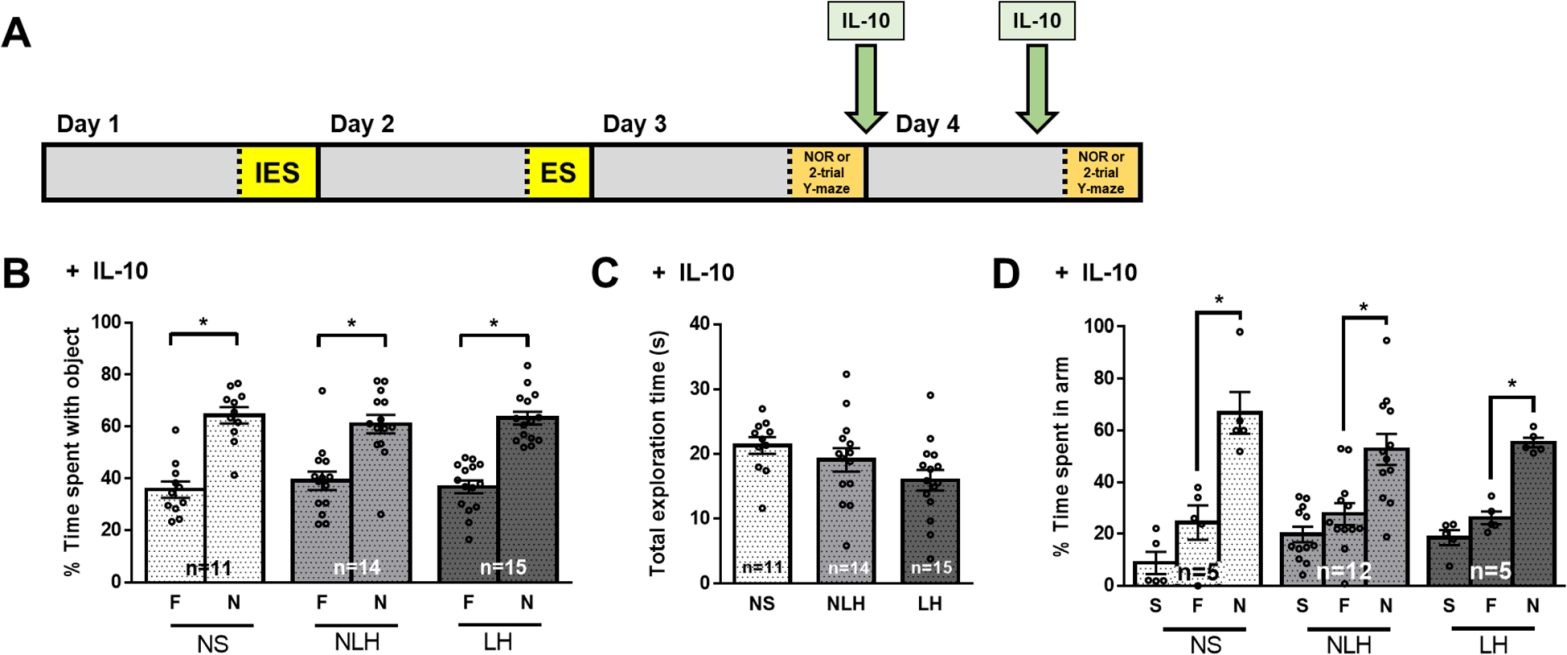
IL-10 reversed LH-dependent recognition and spatial memory impairments. Male mice were subjected or not (NS) to the learned helplessness paradigm and separated into 2 groups: non-learned helpless (NLH) and learned helpless (LH) mice, according to their number of failures out of 30 escapable shock trials, and the next day, learning and memory was assessed. After the cognitive assessments, mice were treated with IL-10 (5 μg/mouse) i.n. and retreated 1 h before the learning and memory assessment on the next day as shown in (**a**). The percentage of a mouse’s total object exploration time spent exploring the familiar (F) and novel (N) objects (**b**) and the total time (s) mice spent engaged in exploration of both the familiar and novel objects combined (**c**) in the novel object recognition test were reported. Each dot represents a mouse. Two-way ANOVA F(2,74)_interaction_ = 0.6239, F(1,74)_treatment_ = 102.8, F(2,74)_condition_ = 4.298 × 10^−14^, Bonferroni post hoc test, *p < 0.05, compared to % time spent with familiar object, bars represent means ± SEM, *n* = 11–15 mice/group. **d** The percentage of a mouse’s total maze exploration time spent exploring the start (S), familiar (F), and novel (N) arms in the two-trial Y-maze test was measured in a different cohort of mice than **b**. Each dot represents a mouse. Two-way ANOVA, F(4,57)_interaction_ = 1.501, F(2,57)_treatment_ = 46.50, F(2,57)_condition_ = 4.036 × 10^−11^, Fisher’s least significant difference test, *p < 0.05, bars represent means ± SEM, *n* = 5–12 mice/group

Since in LH mice microglial activation was reduced by treatment with IL-10 (Fig. 1 f, h–j) and microglial cells contributed to the impaired learning and memory in LH mice (Fig. 3 b–d), we tested if IL-10 administration reverses cognitive impairments in LH mice. Mice were subjected or not to the learned helplessness paradigm and treated with intranasal IL-10, 24 h and 1 h before cognitive assessment (Fig. 5 a). Administration of IL-10 blocked the impairments in novel object recognition and spatial memory in LH mice, whereas IL-10 treatment did not affect novel object recognition or spatial memory of NS and NLH mice (Fig. 5 b–d, Suppl Fig 3A). IL-10 did not seem to affect learned helpless behavior per se as the percent of learned helpless mice after IL-10 treatment was similar to the one of mice after vehicle treatment (Suppl Fig 5A).

These ameliorative effects of intranasal IL-10 treatment on cognitive impairments in LH mice led us to test if IL-10 treatment was also effective in other mouse models that display impaired learning and memory, the mouse model of Fragile X syndrome [*Fmrp1*^−/−^ mice [20];] and mice expressing constitutively active glycogen synthase kinase-3 (GSK3) [GSK3 knock-in mice [38];]. IL-10 treatment reversed novel object recognition impairments in both *Fmrp1*^−/−^ mice (Suppl Fig 5C-F) and GSK3 knock-in mice (Suppl Fig 5G-J). We also tested if IL-10 treatment ameliorated cognitive impairments in female wild-type mice. Similar to wild-type male mice, IL-10 also reversed novel object recognition impairments in female mice (Suppl Fig 6), in both NLH and LH mice as both female NLH and LH mice exhibited novel object recognition impairments.

### IL-10 is sufficient to rescue the NLH-dependent novel object recognition and spatial memory impairments associated with microglial depletion

**Fig. 6:**
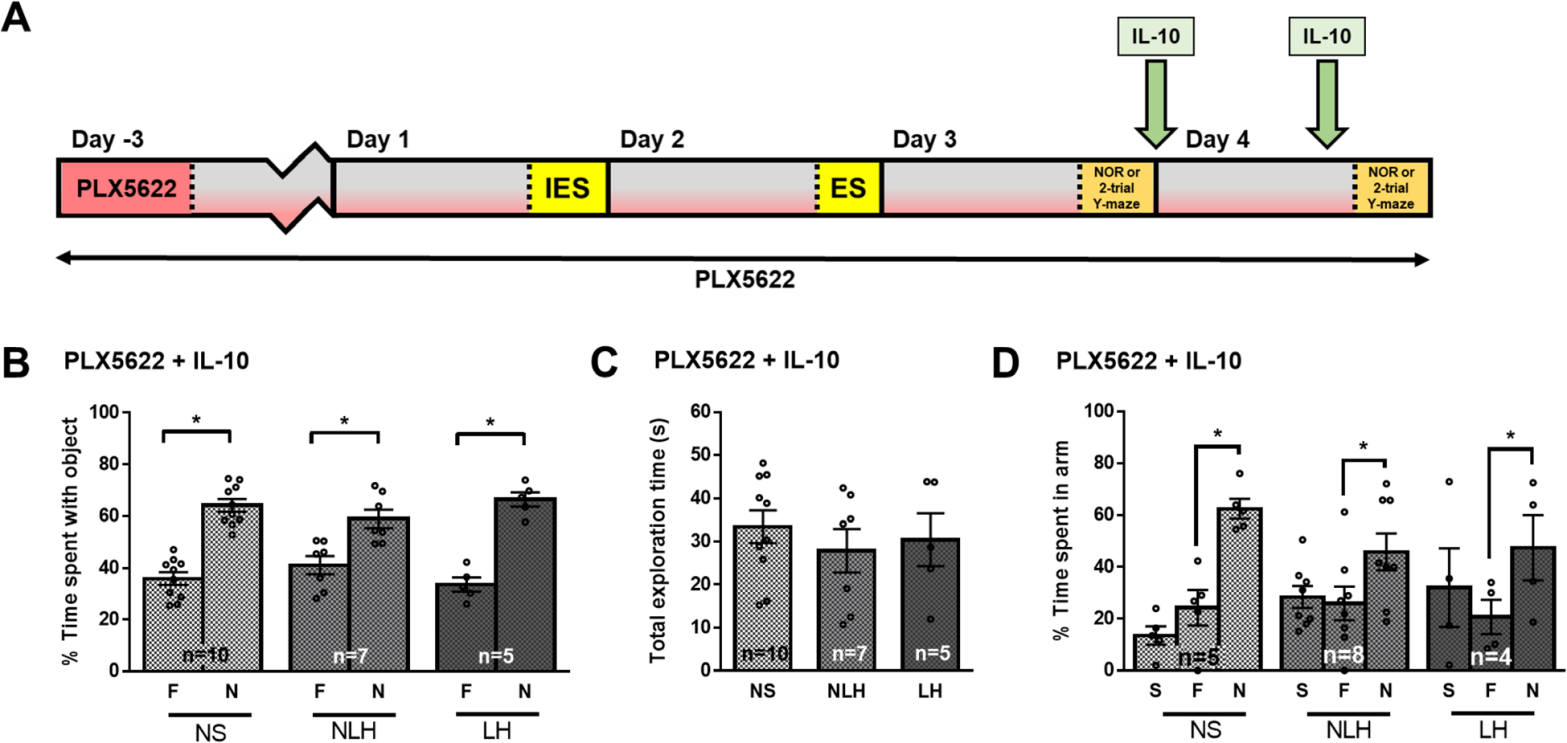
IL-10 reversed the learning and memory impairments induced by microglia depletion in NLH mice. Male mice were fed with the AIN-76A diet supplemented with PLX5622 for 3 days before the induction of the learned helplessness paradigm and were maintained on the diet for the duration of the experiment. Three days after initiating the diet, mice were subjected or not (NS) to the learned helplessness paradigm and separated into 2 groups: non-learned helpless (NLH) and learned helpless (LH) mice, according to their number of failures out of 30 escapable shock trials, and the next day, learning and memory was assessed. After the cognitive assessments, mice were treated with IL-10 (5 μg/mouse) i.n. and retreated 1 h before the learning and memory re-assessment on the next day as shown in (**a**). The percentage of a mouse’s total object exploration time spent exploring the familiar (F) and novel (N) objects (**b**) and the total time (s) mice spent engaged in exploration of both the familiar and novel objects combined (**c**) in the novel object recognition test were reported. Each dot represents a mouse. Two-way ANOVA F(2,38)_interaction_ = 2.918, F(1,38)_treatment_ = 109.4, F(2,38)_condition_ = 1.415 × 10^−14^, Bonferroni post hoc test, *p < 0.05, compared to % time spent with familiar object, bars represent means ± SEM, *n* = 5–10 mice/group. **d** The percentage of a mouse’s total maze exploration time spent exploring the start (S), familiar (F), and novel (N) arms in the two-trial Y-maze test was measured in a different cohort of mice than **b**. Each dot represents a mouse. Two-way ANOVA, F(4,42)_interaction_ = 1.804, F(2,42)_treatment_ = 12.46, F(2,42)_condition_ = 8.528 × 10^−14^, Fisher’s least significant difference test, *p < 0.05, bars represent means ± SEM, *n* = 4–8 mice/group

To determine if amelioration of cognitive impairments by administration of IL-10 actions require microglial cells, microglia were eliminated by PLX5622 treatment followed by exposure to the LH paradigm and IL-10 treatment (Fig. 6 a). IL-10 administration had no effect on novel object recognition and spatial memory of PLX5622-treated NS mice or in PLX5622-treated LH mice since the absence of microglia was sufficient to rescue novel object recognition and spatial memory of LH mice (Fig. 6 b–d, Suppl Fig 3A). However, in microglia-depleted NLH mice, IL-10 rescued impaired novel object recognition and spatial memory, suggesting that in NLH mice, microglial cells might secrete IL-10 to maintain cognitive performances, consistent with data shown in Fig. 1 k. In contrast, in LH mice, the contribution of microglial cells in regulating learning and memory is deleterious, and IL-10 improved learning and memory whether or not microglial cells are present, suggesting that either microglial cells adopt a pro-inflammatory profile in LH mice, and the addition of the anti-inflammatory cytokine IL-10 attenuates the microglial inflammatory response, or that IL-10 acts independently of microglial cells.

### Depletion of STAT3 abolished the beneficial effect of IL-10

**Fig. 7:**
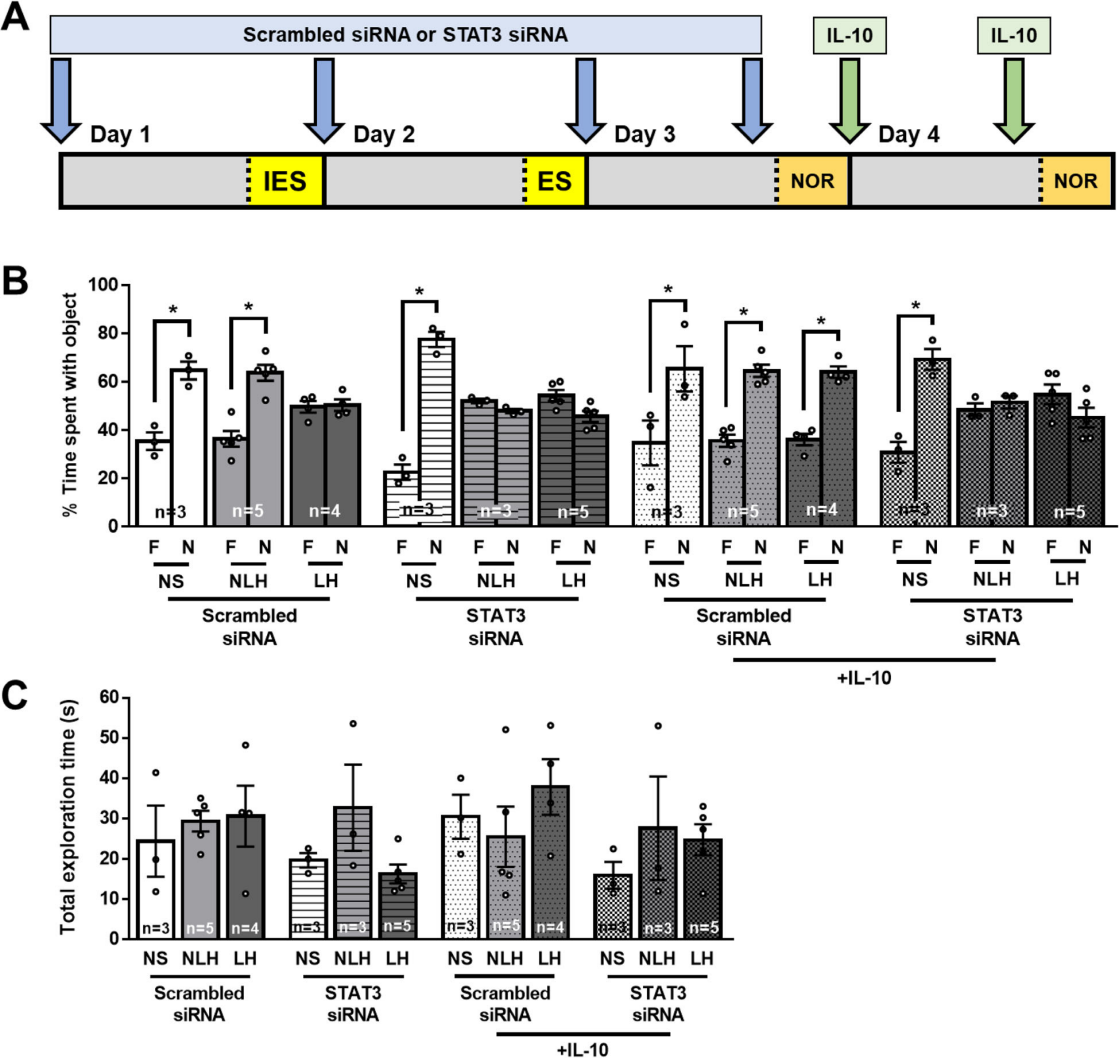
STAT3 knockdown impairs novel object recognition in NLH mice and blocked IL-10 effects IL-10 to reverse impairments. Male mice were treated intranasally with a scrambled siRNA control or STAT3 siRNA (10 μg/mouse) 1 day prior to the induction of the learned helplessness paradigm and treated every day thereafter. Mice were subjected or not (NS) to the learned helplessness paradigm and separated into 2 groups: non-learned helpless (NLH) and learned helpless (LH) mice, according to their number of failures out of 30 escapable shock trials, and the next day, novel object recognition was assessed. After the cognitive assessments, mice were treated with IL-10 (5 μg/mouse) i.n. and retreated 1 h before the second novel object recognition re-assessment on the next day as shown in (**a**). The percentage of a mouse’s total object exploration time spent exploring the familiar (F) and novel (N) objects in scrambled siRNA and STAT3 siRNA treated mice and in IL-10-treated, scrambled siRNA- and STAT3 siRNA-treated mice (**b**) as well as the total time (s) mice spent engaged in exploration of both the familiar and novel objects combined (**c**) in the novel object recognition test were reported. Each dot represents a mouse. Two-way ANOVA F(14,68)_interaction_ = 10.42, F(7,68)_treatment_ = 24.11, F(2,68)_condition_ = 3.088 × 10^−13^, Bonferroni post hoc test, *p < 0.05, compared to % time spent with familiar object, bars represent means ± SEM, *n* = 3–5 mice/group

To determine if STAT3 contributes to the IL-10 pro-cognitive effects, since STAT3 mediates IL-10 signaling, we knocked down STAT3 in the brain (Fig. 7 a), using STAT3 siRNA delivered intranasally, which we previously showed was sufficient to knock down protein expression in the hippocampus by 75% [44]. Seventy-four percent depletion of STAT3 was confirmed by immunohistochemistry (Suppl Fig 7A-H). As expected, knockdown of STAT3 induced novel object recognition impairment in NLH mice (Fig. 7 b, c, and Suppl Fig 7I), similarly to the microglial depletion by PLX5622. We found that depletion of STAT3 blocked the rescue by IL-10 of the novel object recognition impairment of LH mice, whereas delivery of a scrambled siRNA did not affect IL-10 effects, demonstrating that STAT3 was required to mediate IL-10 effects in LH mice (Fig. 7 b, c, and Suppl Fig 7I). We also found that the impairment of novel object recognition in NLH mice could not be rescued by IL-10, consistent with the idea that in NLH mice, microglial cells might secrete IL-10 to maintain cognitive performances, and that in the absence of STAT3, IL-10 is unable to mediate its beneficial cognitive effects. However, depletion of STAT3 in NS mice had no effect on novel object recognition, suggesting that IL-10 is critical in the stress response, and that other pathways contribute to learning and memory in non-shocked mice, and might compensate for the absence of STAT3.

## Discussion

The mechanisms of cognitive impairments induced by stress and those associated with depression remain poorly understood. We found that the induction of learned helplessness in mice was associated with impaired novel object recognition and spatial memory, decreased dendritic spine density, increased microglial activation, and slightly reduced IL-10-producing microglia. Microglial cells were found to be involved in the novel object recognition and spatial memory impairments because depleting microglia prevented the development of cognitive impairments in learned helpless mice. We further identified the IL-10/STAT3 pathway as a potential mechanism promoting novel object recognition and spatial memory after stress.

Consistent with previous reports, microglial cells were activated in response to stress, and this correlated with reduced dendritic spine density [61]. Microglial cells are able to phagocyte synapses, and therefore shape neuronal circuits [16, 51, 59]. Indeed, resting microglial cells extend and retract their processes to actively survey their environment, including making contacts with neurons [57]. This is thought to have beneficial consequences, such as eliminating ischemic synapses and refining the wiring of neurons [12]. We found that microglial depletion enhances learning and memory after stress. However, it is difficult to detangle if the improvement of learning and memory after PLX5622 could also result from an amelioration of depressive-like behavior, as PLX5622 induces resilience to learned helplessness induction. Nevertheless, it is noticeable that PLX5622 alters learning and memory in resilient mice while ameliorating learning and memory in susceptible mice, uncovering the complex role of microglial cells in learning and memory after stress. In pathological conditions, microglia often adopt an amoeboid phenotype and migrate to sites of injury where they release factors that have been reported to result in either neuroprotection or neurotoxicity [8, 56]. Based on our findings, one of these factors appears to be IL-10. We found that after stress, microglial cells from LH mice express slightly less IL-10 than microglia from NLH mice. This corroborates findings that homeostatic and disease-related microglia may be two distinct populations [22]. Consistent with this, IL-10 levels have been shown to be diminished in depression [17], and a defective anti-inflammatory IL-10 pathway has been associated with resistance to antidepressant treatments [55]. This suggests that some microglial cells might have anti-inflammatory properties in response to stress, participating in the promotion of learning and memory in stressed mice, whereas in stressed LH mice, activated microglial cells acquire an inflammatory phenotype, leading to a reduction of the beneficial action of IL-10 on learning and memory. It is also possible that a new population of microglial cells is induced in LH mice that directly impair learning and memory. To reinforce the idea that microglia are critical in maintaining learning and memory after stress, we found that mice that received foot-shocks but were resilient to learned helplessness induction exhibited cognitive impairments and dampening of dendritic spine density after microglial depletion (Suppl Fig 2D-F). This was rescued by the administration of IL-10, suggesting that (1) microglia are required for learning and memory in stress conditions, even if the mice are resilient, and (2) the microglial cells in NLH mice produce the required IL-10 to maintain learning and memory. Whether the loss of IL-10 expression occurs in selective populations of microglia or results from the induction of a novel population of disease-related microglia in which the production of IL-10 is inhibited, or both, remains to be determined. It is also possible that IL-10 is produced by other cells in the brain than microglial cells but that IL-10 production by other cells requires signaling from the microglia. IL-10 might have direct effects on microglia to prevent them from phagocytosing synapses [49] and/or IL-10 might also act directly on neurons to increase synapse formation [34, 63]. Furthermore, the mechanisms whereby IL-10 promotes dendritic spine density remain to be elucidated. It was reported that STAT3 is involved in synapse formation [54], so IL-10 might promote dendritic spine density by activating STAT3. We showed that STAT3 mediates the behavioral effect of IL-10, as depletion of STAT3 was sufficient to block IL-10 rescue of novel object recognition and spatial memory impairments in LH mice. This adds to the literature showing that STAT3 is important to mediate NMDAR-LTD to modulate synaptic plasticity [41]. Therefore, whether this is the mechanism whereby IL-10 promotes learning and memory in learned helpless mice remains to be further studied. However, it is important to note that depletion of STAT3 in NLH mice but not NS mice also impairs learning and memory, confirming that STAT3 promotes learning and memory in response to stress and favors resilience. Other pathways inducing STAT3 activation might also contribute to learning and memory after stress, but this will need further investigations.

The reduction in IL-10 level or a blockade in IL-10 signaling might in part also explain the puzzling findings that pro-inflammatory cytokines are required at physiological levels to maintain synaptic plasticity and proper neuronal network functioning [6, 13], yet excess levels of pro-inflammatory cytokines are associated with impairments of learning and memory [16, 37]. It is indeed possible that, in the presence of high levels of pro-inflammatory cytokines, IL-10 production or its actions are suppressed, abolishing the beneficial effects of IL-10 on learning and memory, rather than pro-inflammatory cytokines altering neuronal circuits directly. It is noticeable that IL-10 exhibited pro-cognitive effects also in transgenic mice such as *Fmrp*1^−/−^ (FX) and GSK3 knock-in mice without stress, suggesting that similar pathways contribute to learning and memory impairments in these mice as in LH mice, and that IL-10 is able to reverse these effects, pointing toward GSK3 action in impairing learning and memory in FX mice, as previously reported [20, 28]. However, further experiments will be needed to determine the mechanism whereby IL-10 improves learning and memory in FX and GSK3 knock-in mice.

## Conclusions

**Fig. 8:**
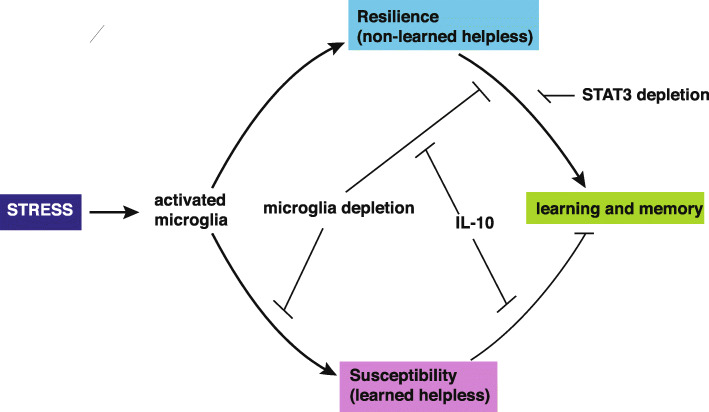
Schematic representation of the role of microglial cells and IL-10 in learning and memory deficits associated with stress resilience and susceptibility

Altogether our findings show that IL-10 provides pro-cognitive actions in learned helpless mice or mice with cognitive impairments possibly associated with microglial dysfunction affecting synapse balance (Fig. 8), opening potential new therapeutic avenues to correct learning and memory deficits.

## Supplementary information

**Additional file 1: Supplemental Figure 1.** IL-10 promotes activation of STAT3 in the hippocampus, cerebral prefrontal cortex and cerebellum. (A) Gating strategy used for the analysis of IL-10+CD45Int cells by flow cytometry after learned helplessness. (B) Male mice were treated intranasally with either vehicle (Veh.) or IL-10 (5 μg/mouse) for 1 h. Phospho-Tyr^705^-STAT3 (PYSTAT3) and STAT3 proteins were immunoblotted in the hippocampus, cerebral prefrontal cortex and cerebellum. Membranes were reblotted for b-actin to ensure proper loading. Quantification of the samples on the top were represented as the ratio of PYSTAT3/STAT3 in each brain region, and a representative image of the western blot was shown in the bottom. Mann-Whitney test, U=0, 4, and 3, *p<0.05 compared to vehicle-treated mice, bars represent means ± SEM, n = 6-8. **Supplemental Figure 2**. The decrease of hippocampal CA1 and CA3 dendritic spine density in stressed mice was abolished by IL-10 treatment. Male wild-type mice were subjected or not (NS) to the learned helplessness paradigm and separated into 2 groups: non-learned helpless (NLH), and learned helpless (LH) mice, according to their number of failures out of 30 escapable shock trials and were treated or not with vehicle (Veh.) or IL-10 (5 μg/mouse) 24 h and 1 h prior to sacrifice. (A) Representative image of dendritic spines on apical dendrites radiating from granule cells within the dentate gyrus (DG) of a NLH mouse. Arrows point to the actual dendritic spines that were recorded. (B) Dendritic spine density was calculated as an average per animal (# spines/μm) over 80 μm of total analyzed apical dendrite length (20 μm from the cell body) extending from granule cells within the molecular layer of the dentate gyrus and pyramidal cells within the stratum radiatum of the CA1 (B) and CA3 (C) regions of the dorsal hippocampus. Each dot represents a mouse. Two-way ANOVA F(2,33)_interaction_=15.52, F(1,33)_treatment_=10.36, F(2,33)_condition_=6.814, Bonferroni post-hoc test, *p<0.05, bars represent mean ± SEM, n=5-7 mice/group. Male mice were fed with the AIN-76A diet supplemented with PLX5622 for 3 days before the induction of the learned helplessness paradigm and were maintained on the diet for the duration of the experiment. 3 days after initiating the diet, mice were subjected or not (NS) to the learned helplessness paradigm and separated into 2 groups: non-learned helpless (NLH), and learned helpless (LH) mice, according to their number of failures out of 30 escapable shock trials, and the next day learning and memory was assessed. After the cognitive assessments, mice were treated with IL-10 (5 μg/mouse) i.n. and retreated 1 h before the learning and memory re-assessment on the next day as shown in Fig 6. Dendritic spine densities in (D) CA1, (E) CA3, and (F) dentate gyrus were calculated. Each dot represents a mouse. Two-way ANOVA F(2,24)_interaction_=44.31, F(1,24)_treatment_=17.43, F(2,24)_condition_=12.09, Bonferroni post-hoc test, *p<0.05, bars represent mean ± SEM, n=3-8 mice/group. **Supplemental Figure 3**. The impairment seen in LH mice was not the result of anxiety or inability to recognize the cues. (A) Male mice were fed or not with the AIN-76A diet (control diet), the AIN-76A diet supplemented with PLX5622 for 3 days before the induction of the learned helplessness paradigm and were maintained on the diet for the duration of the experiment. 3 days after initiating the diet, mice were subjected or not (NS) to the learned helplessness paradigm and separated into 2 groups: non-learned helpless (NLH), and learned helpless (LH) mice, according to their number of failures out of 30 escapable shock trials, and the next day learning and memory was assessed. After the cognitive assessments, mice were treated with IL-10 (5 μg/mouse) i.n. and retreated 1 h before the novel object recognition assessment on the next day. The discrimination index is represented. Each dot represents a mouse. Two-way ANOVA, F(2,82)_interaction_=4.627, F(1,82)_treatment_=2.914, F(2,82)_condition_=2.991, Bonferroni post-hoc test, *p<0.05, bars represent mean ± SEM, n = 11-18 mice/group; Two-way ANOVA, F(4,57)_interaction_=4.832, F(2,57)_treatment_=1.984, F(2,57)_condition_=3.043, Bonferroni post-hoc test, *p<0.05, bars represent mean ± SEM, n=4-12 mice/group. Male mice were fed or not with the AIN-76A diet supplemented with PLX5622 for 3 days before the induction of the learned helplessness paradigm and were maintained on the diet for the duration of the experiment. 3 days after initiating the diet, mice were subjected or not (NS) to the learned helplessness paradigm and separated into 2 groups: non-learned helpless (NLH), and learned helpless (LH) mice, according to their number of failures out of 30 escapable shock trials, and the next day learning and memory was assessed as described in B and E. After the first cognitive assessment (C), mice were treated with IL-10 (5 μg/mouse) i.n. and retreated 1 h before the second spatial memory re-assessment on the next day. The percentage of a mouse’s total maze exploration time spent exploring the start (S), familiar (F), and novel (N) arms using a modified 1-min inter-trial interval (ITI) in the two-trial Y-maze test was measured in different cohorts of mice receiving (F-G) or not (C-D) PLX5622. Each dot represents a mouse. One-way ANOVA, F(8,24)=19.14 (C), F(8,18)=18.61 (D), F(8,27)=27.59 (F), and F(8,21)=14.89 (G), Fisher’s least significant difference test, *p<0.05, bars represent means ± SEM, n=3-5 mice/group. **Supplemental Figure 4**. PLX5622 deplete ~90% of microglia in the hippocampus. Male mice were fed with the AIN-76A diet or AIN-76A diet supplemented with PLX5622 for 3 days. Brains were immunostained for Iba1 and DAPI. (A) Number of DG Iba1^+^ microglia per 250,000 μm^2^ sampling area. Each dot represents a mouse. Mann-Whitney test, U=0, *p<0.05, bars represent means ± SEM, n=3 mice/group. (B-C) Representative images of Iba1 immunofluorescence in the dentate gyrus of mice receiving the (B) AIN-76A diet or (C) AIN-76A diet supplemented with PLX5622. (D) Male mice were fed with the AIN-76A diet or AIN-76A diet supplemented with PLX5622 for 3 days before the induction of the learned helplessness paradigm and mice were maintained on the diet for the duration of the experiment. 3 days after initiating the diet, mice were subjected to the learned helplessness paradigm and the number of failures to escape was recorded. Each dot represents a mouse. Mann-Whitney test, U=88, *p<0.05, bars represent mean ± SEM, n=9-29 mice/group. **Supplemental Figure 5**. IL-10 restored the novel object recognition deficit of *Fmr1*^–/–^ and GSK3α/β^21A/21A/9A/9A^ knock-in mice. (A) Male wild-type mice were subjected to the learned helplessness paradigm and separated into 2 groups: non-learned helpless (NLH), and learned helpless (LH) mice, according to their number of failures out of 30 escapable shock trials and LH mice were treated with vehicle or IL-10 (5 μg/mouse) 24 h and 1 h prior to retesting the number of failures. Mann-Whitney test, U=17.50, p=0.2211, bars represent means ± SEM, n=7-8 mice/group. Novel object recognition was assessed in male littermate wild-type (WT) and *Fmr1*^–/–^ (FX) mice or GSK3α/β^21A/21A/9A/9A^ knock-in (GSK3 DKI) mice, and treated intranasally with IL-10 (5 μg/mouse) after the first behavioral assessment and 1 h before the second behavioral re-assessment as shown in (B). The percentage of a mouse’s total object exploration time spent exploring the familiar (F) and novel (N) objects (C-D, G-H) and the total time (s) mice spent engaged in exploration of both the familiar and novel objects combined (E-F, I-J) in the novel object recognition task was reported for the FX mice (C-F) or the GSK3 DKI mice (G-J). Each dot represents a mouse. One-way ANOVA, F(3,72)=11.56 (C), F(3,60)=29.64 (D), F(3,46)=4.436 (G), F(3,18)=12.42 (H), Bonferroni post-hoc test, *p<0.05, compared to % time spent with familiar object, Student’s t test t=2.629 (F), bars represent means ± SEM, n=14-21 (C-F), n=4-16 (G-J). (B) Male WT mice were subjected to the learned helplessness paradigm. LH mice were then treated i.n. with vehicle (Veh.) or IL-10 (5 μg/mouse) 24 h prior to reassessing learned helplessness the next day with a second round of 30 escapable shock trials to determine if IL-10 induces a higher recovery to learned helplessness than vehicle.. The number of escape failures is reported. Each dot represents a mouse. Mann-Whitney test, U=17.5, *p<0.05, bars represent mean ± SEM, n=7-8 mice/group. **Supplemental Figure 6**. Novel object recognition deficit was observed in both NLH and LH female mice and the novel object recognition in both NLH and LH female mice was restored by IL-10. Female wild-type mice were subjected or not (NS) to the learned helplessness paradigm and separated into 2 groups: non-learned helpless (NLH), and learned helpless (LH) mice, according to their number of failures out of 30 escapable shock trials and the next day learning and memory was assessed. After the first cognitive assessment, mice were treated with IL-10 (5 μg/mouse) i.n. and retreated 1 h before the learning and memory re-assessment on the next day as shown in (A). The percentage of a mouse’s total object exploration time spent exploring the familiar (F) and novel (N) objects (B-C) and the total time (s) mice spent engaged in exploration of both the familiar and novel objects combined (D-E) in the novel object recognition task were reported. Each dot represents a mouse. One-way ANOVA F(5,94)=3.385 (B), F(5,44)=19.94 (C), Bonferroni post-hoc test, *p<0.05, compared to % time spent with familiar object, bars represent means ± SEM, n=5-24 mice/group. **Supplemental Figure 7**. STAT3 expression was reduced by ~74% in the hippocampus after i.n. treatment with STAT3 siRNA. Mice were treated intranasally once daily with a scrambled siRNA control or STAT3 siRNA (10 μg/mouse) 1 day prior to the induction of the learned helplessness paradigm and treated every day thereafter. Mice were subjected or not (NS) to the learned helplessness paradigm and separated into 2 groups: non-learned helpless (NLH), and learned helpless (LH) mice, according to their number of failures out of 30 escapable shock trials. One hour after the last treatment, mice were perfused and brains were collected for immunostaining for STAT3 and DAPI. (A-F) Representative images of STAT3 immunofluorescence in the hippocampus of scrambled siRNA-treated NS (A), NLH (B), and LH (C) mice, and STAT3 siRNA-treated NS (D), NLH (E), and LH (F) mice from the region delimited by the square in (G). (H) Number of STAT3^+^ focal maxima per 250,000 μm^2^ sampling area within the hippocampus was quantified. Data from NS, NLH, and LH mice was pooled under control and experimental conditions. Each dot represents a mouse. Mann-Whitney test, U=0, *p<0.05, bars represent means ± SEM, n=5/group. (I) Discrimination index in the novel object recognition. Each dot represents a mouse. Two-way ANOVA, F(6,34)_interaction_=4.392, F(3,34)_treatment_=3.538, F(2,34)_condition_=22.82, Bonferroni post-hoc test, *p<0.05, bars represent mean ± SEM, n=3-5 mice/group.

**Additional file 2: Supplemental Table 1:** *p*-values for each figure.

## Data Availability

The data and some materials, but not all (we have a MTA agreement with Plexxikon), will be available upon request.

## Abbreviations

ES: Escapable foot-shocks
FX: Fragile X
GM-CSF: Granulocyte-macrophage colony-stimulating factor
GSK3: Glycogen synthase kinase-3
IES: Inescapable foot-shocks
IL-: Interleukin
ITI: Inter-trial interval
LH: Learned helpless
LPS: Lipopolysaccharide
LTP: Long-term potentiation
MDD: Major depressive disorder
M-CSF: Macrophage colony-stimulating factor
NLH: Non-learned helpless
NOR: Novel object recognition
NS: Non-shocked
STAT3: Signal transducer and activator of transcription 3
