# Supplementary figures and images for: Anti-inflammatory IL-10 administration rescues depression-associated learning and memory deficits in mice
Source: J Neuroinflammation. 2020 Aug 22;17:246. doi: 10.1186/s12974-020-01922-1 (PMC7443292; doi:10.1186/s12974-020-01922-1)

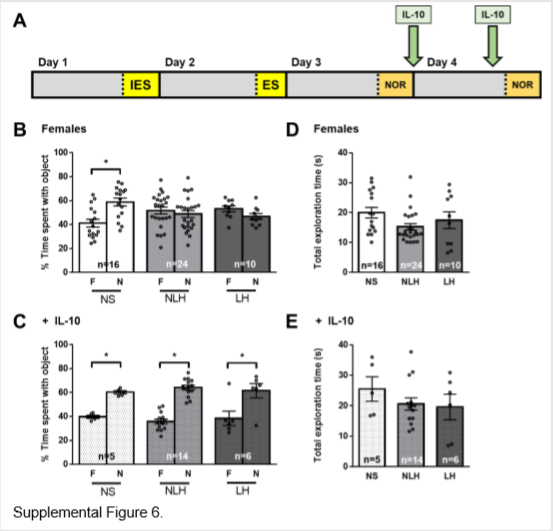

Supplement: Supplementary file 1 — Additional file 1: Supplemental Figure 1. IL-10 promotes activation of STAT3 in the hippocampus, cerebral prefrontal cortex and cerebellum. (A) Gating strategy used for the analysis of IL-10+CD45Int cells by flow cytometry after learned helplessness. (B) Male mice were treated intranasally with either vehicle (Veh.) or IL-10 (5 μg/mouse) for 1 h. Phospho-Tyr705-STAT3 (PYSTAT3) and STAT3 proteins were immunoblotted in the hippocampus, cerebral prefrontal cortex and cerebellum. Membranes were reblotted for b-actin to ensure proper loading. Quantification of the samples on the top were represented as the ratio of PYSTAT3/STAT3 in each brain region, and a representative image of the western blot was shown in the bottom. Mann-Whitney test, U=0, 4, and 3, *p<0.05 compared to vehicle-treated mice, bars represent means ± SEM, n = 6-8. Supplemental Figure 2. The decrease of hippocampal CA1 and CA3 dendritic spine density in stressed mice was abolished by IL-10 treatment. Male wild-type mice were subjected or not (NS) to the learned helplessness paradigm and separated into 2 groups: non-learned helpless (NLH), and learned helpless (LH) mice, according to their number of failures out of 30 escapable shock trials and were treated or not with vehicle (Veh.) or IL-10 (5 μg/mouse) 24 h and 1 h prior to sacrifice. (A) Representative image of dendritic spines on apical dendrites radiating from granule cells within the dentate gyrus (DG) of a NLH mouse. Arrows point to the actual dendritic spines that were recorded. (B) Dendritic spine density was calculated as an average per animal (# spines/μm) over 80 μm of total analyzed apical dendrite length (20 μm from the cell body) extending from granule cells within the molecular layer of the dentate gyrus and pyramidal cells within the stratum radiatum of the CA1 (B) and CA3 (C) regions of the dorsal hippocampus. Each dot represents a mouse. Two-way ANOVA F(2,33)interaction=15.52, F(1,33)treatment=10.36, F(2,33)condition=6.814, Bonferroni [file 12974_2020_1922_MOESM1_ESM.zip › Fgure S6.PNG]

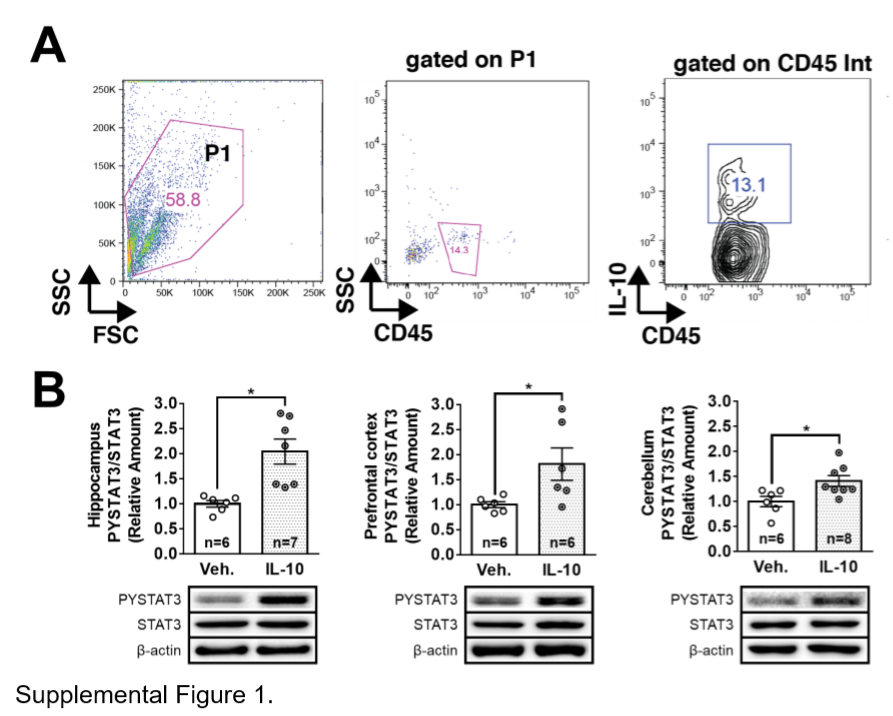

Supplement: Supplementary file 1 — Additional file 1: Supplemental Figure 1. IL-10 promotes activation of STAT3 in the hippocampus, cerebral prefrontal cortex and cerebellum. (A) Gating strategy used for the analysis of IL-10+CD45Int cells by flow cytometry after learned helplessness. (B) Male mice were treated intranasally with either vehicle (Veh.) or IL-10 (5 μg/mouse) for 1 h. Phospho-Tyr705-STAT3 (PYSTAT3) and STAT3 proteins were immunoblotted in the hippocampus, cerebral prefrontal cortex and cerebellum. Membranes were reblotted for b-actin to ensure proper loading. Quantification of the samples on the top were represented as the ratio of PYSTAT3/STAT3 in each brain region, and a representative image of the western blot was shown in the bottom. Mann-Whitney test, U=0, 4, and 3, *p<0.05 compared to vehicle-treated mice, bars represent means ± SEM, n = 6-8. Supplemental Figure 2. The decrease of hippocampal CA1 and CA3 dendritic spine density in stressed mice was abolished by IL-10 treatment. Male wild-type mice were subjected or not (NS) to the learned helplessness paradigm and separated into 2 groups: non-learned helpless (NLH), and learned helpless (LH) mice, according to their number of failures out of 30 escapable shock trials and were treated or not with vehicle (Veh.) or IL-10 (5 μg/mouse) 24 h and 1 h prior to sacrifice. (A) Representative image of dendritic spines on apical dendrites radiating from granule cells within the dentate gyrus (DG) of a NLH mouse. Arrows point to the actual dendritic spines that were recorded. (B) Dendritic spine density was calculated as an average per animal (# spines/μm) over 80 μm of total analyzed apical dendrite length (20 μm from the cell body) extending from granule cells within the molecular layer of the dentate gyrus and pyramidal cells within the stratum radiatum of the CA1 (B) and CA3 (C) regions of the dorsal hippocampus. Each dot represents a mouse. Two-way ANOVA F(2,33)interaction=15.52, F(1,33)treatment=10.36, F(2,33)condition=6.814, Bonferroni [file 12974_2020_1922_MOESM1_ESM.zip › Figure S1.PNG]

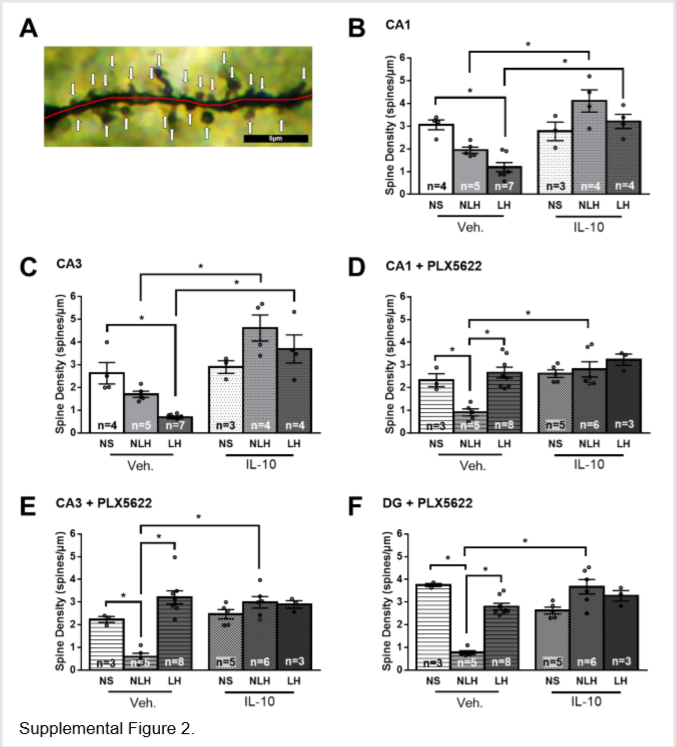

Supplement: Supplementary file 1 — Additional file 1: Supplemental Figure 1. IL-10 promotes activation of STAT3 in the hippocampus, cerebral prefrontal cortex and cerebellum. (A) Gating strategy used for the analysis of IL-10+CD45Int cells by flow cytometry after learned helplessness. (B) Male mice were treated intranasally with either vehicle (Veh.) or IL-10 (5 μg/mouse) for 1 h. Phospho-Tyr705-STAT3 (PYSTAT3) and STAT3 proteins were immunoblotted in the hippocampus, cerebral prefrontal cortex and cerebellum. Membranes were reblotted for b-actin to ensure proper loading. Quantification of the samples on the top were represented as the ratio of PYSTAT3/STAT3 in each brain region, and a representative image of the western blot was shown in the bottom. Mann-Whitney test, U=0, 4, and 3, *p<0.05 compared to vehicle-treated mice, bars represent means ± SEM, n = 6-8. Supplemental Figure 2. The decrease of hippocampal CA1 and CA3 dendritic spine density in stressed mice was abolished by IL-10 treatment. Male wild-type mice were subjected or not (NS) to the learned helplessness paradigm and separated into 2 groups: non-learned helpless (NLH), and learned helpless (LH) mice, according to their number of failures out of 30 escapable shock trials and were treated or not with vehicle (Veh.) or IL-10 (5 μg/mouse) 24 h and 1 h prior to sacrifice. (A) Representative image of dendritic spines on apical dendrites radiating from granule cells within the dentate gyrus (DG) of a NLH mouse. Arrows point to the actual dendritic spines that were recorded. (B) Dendritic spine density was calculated as an average per animal (# spines/μm) over 80 μm of total analyzed apical dendrite length (20 μm from the cell body) extending from granule cells within the molecular layer of the dentate gyrus and pyramidal cells within the stratum radiatum of the CA1 (B) and CA3 (C) regions of the dorsal hippocampus. Each dot represents a mouse. Two-way ANOVA F(2,33)interaction=15.52, F(1,33)treatment=10.36, F(2,33)condition=6.814, Bonferroni [file 12974_2020_1922_MOESM1_ESM.zip › Figure S2.PNG]

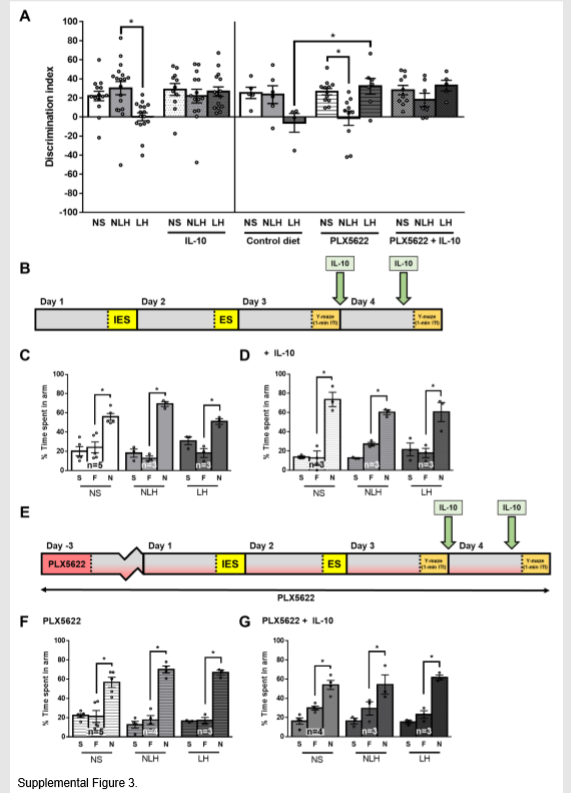

Supplement: Supplementary file 1 — Additional file 1: Supplemental Figure 1. IL-10 promotes activation of STAT3 in the hippocampus, cerebral prefrontal cortex and cerebellum. (A) Gating strategy used for the analysis of IL-10+CD45Int cells by flow cytometry after learned helplessness. (B) Male mice were treated intranasally with either vehicle (Veh.) or IL-10 (5 μg/mouse) for 1 h. Phospho-Tyr705-STAT3 (PYSTAT3) and STAT3 proteins were immunoblotted in the hippocampus, cerebral prefrontal cortex and cerebellum. Membranes were reblotted for b-actin to ensure proper loading. Quantification of the samples on the top were represented as the ratio of PYSTAT3/STAT3 in each brain region, and a representative image of the western blot was shown in the bottom. Mann-Whitney test, U=0, 4, and 3, *p<0.05 compared to vehicle-treated mice, bars represent means ± SEM, n = 6-8. Supplemental Figure 2. The decrease of hippocampal CA1 and CA3 dendritic spine density in stressed mice was abolished by IL-10 treatment. Male wild-type mice were subjected or not (NS) to the learned helplessness paradigm and separated into 2 groups: non-learned helpless (NLH), and learned helpless (LH) mice, according to their number of failures out of 30 escapable shock trials and were treated or not with vehicle (Veh.) or IL-10 (5 μg/mouse) 24 h and 1 h prior to sacrifice. (A) Representative image of dendritic spines on apical dendrites radiating from granule cells within the dentate gyrus (DG) of a NLH mouse. Arrows point to the actual dendritic spines that were recorded. (B) Dendritic spine density was calculated as an average per animal (# spines/μm) over 80 μm of total analyzed apical dendrite length (20 μm from the cell body) extending from granule cells within the molecular layer of the dentate gyrus and pyramidal cells within the stratum radiatum of the CA1 (B) and CA3 (C) regions of the dorsal hippocampus. Each dot represents a mouse. Two-way ANOVA F(2,33)interaction=15.52, F(1,33)treatment=10.36, F(2,33)condition=6.814, Bonferroni [file 12974_2020_1922_MOESM1_ESM.zip › Figure S3.PNG]

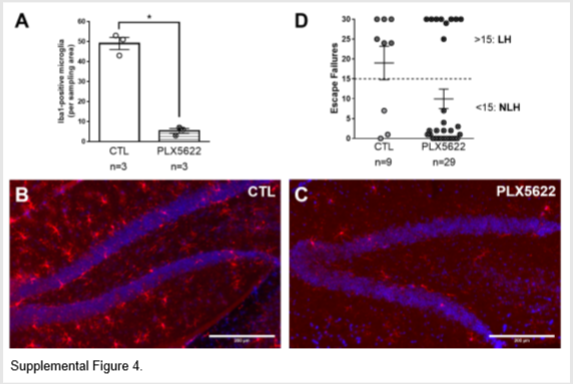

Supplement: Supplementary file 1 — Additional file 1: Supplemental Figure 1. IL-10 promotes activation of STAT3 in the hippocampus, cerebral prefrontal cortex and cerebellum. (A) Gating strategy used for the analysis of IL-10+CD45Int cells by flow cytometry after learned helplessness. (B) Male mice were treated intranasally with either vehicle (Veh.) or IL-10 (5 μg/mouse) for 1 h. Phospho-Tyr705-STAT3 (PYSTAT3) and STAT3 proteins were immunoblotted in the hippocampus, cerebral prefrontal cortex and cerebellum. Membranes were reblotted for b-actin to ensure proper loading. Quantification of the samples on the top were represented as the ratio of PYSTAT3/STAT3 in each brain region, and a representative image of the western blot was shown in the bottom. Mann-Whitney test, U=0, 4, and 3, *p<0.05 compared to vehicle-treated mice, bars represent means ± SEM, n = 6-8. Supplemental Figure 2. The decrease of hippocampal CA1 and CA3 dendritic spine density in stressed mice was abolished by IL-10 treatment. Male wild-type mice were subjected or not (NS) to the learned helplessness paradigm and separated into 2 groups: non-learned helpless (NLH), and learned helpless (LH) mice, according to their number of failures out of 30 escapable shock trials and were treated or not with vehicle (Veh.) or IL-10 (5 μg/mouse) 24 h and 1 h prior to sacrifice. (A) Representative image of dendritic spines on apical dendrites radiating from granule cells within the dentate gyrus (DG) of a NLH mouse. Arrows point to the actual dendritic spines that were recorded. (B) Dendritic spine density was calculated as an average per animal (# spines/μm) over 80 μm of total analyzed apical dendrite length (20 μm from the cell body) extending from granule cells within the molecular layer of the dentate gyrus and pyramidal cells within the stratum radiatum of the CA1 (B) and CA3 (C) regions of the dorsal hippocampus. Each dot represents a mouse. Two-way ANOVA F(2,33)interaction=15.52, F(1,33)treatment=10.36, F(2,33)condition=6.814, Bonferroni [file 12974_2020_1922_MOESM1_ESM.zip › Figure S4.PNG]

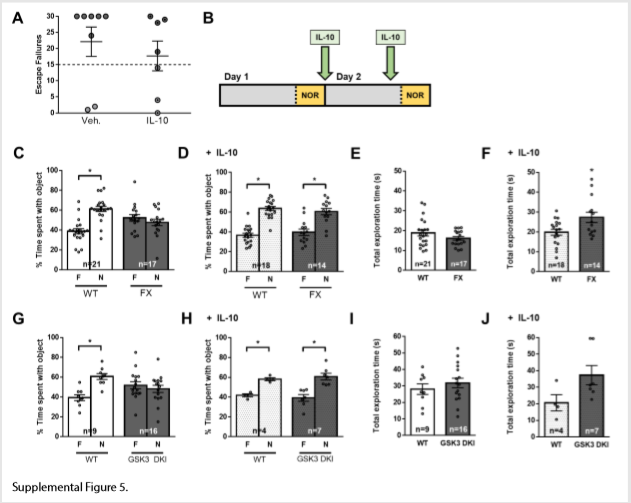

Supplement: Supplementary file 1 — Additional file 1: Supplemental Figure 1. IL-10 promotes activation of STAT3 in the hippocampus, cerebral prefrontal cortex and cerebellum. (A) Gating strategy used for the analysis of IL-10+CD45Int cells by flow cytometry after learned helplessness. (B) Male mice were treated intranasally with either vehicle (Veh.) or IL-10 (5 μg/mouse) for 1 h. Phospho-Tyr705-STAT3 (PYSTAT3) and STAT3 proteins were immunoblotted in the hippocampus, cerebral prefrontal cortex and cerebellum. Membranes were reblotted for b-actin to ensure proper loading. Quantification of the samples on the top were represented as the ratio of PYSTAT3/STAT3 in each brain region, and a representative image of the western blot was shown in the bottom. Mann-Whitney test, U=0, 4, and 3, *p<0.05 compared to vehicle-treated mice, bars represent means ± SEM, n = 6-8. Supplemental Figure 2. The decrease of hippocampal CA1 and CA3 dendritic spine density in stressed mice was abolished by IL-10 treatment. Male wild-type mice were subjected or not (NS) to the learned helplessness paradigm and separated into 2 groups: non-learned helpless (NLH), and learned helpless (LH) mice, according to their number of failures out of 30 escapable shock trials and were treated or not with vehicle (Veh.) or IL-10 (5 μg/mouse) 24 h and 1 h prior to sacrifice. (A) Representative image of dendritic spines on apical dendrites radiating from granule cells within the dentate gyrus (DG) of a NLH mouse. Arrows point to the actual dendritic spines that were recorded. (B) Dendritic spine density was calculated as an average per animal (# spines/μm) over 80 μm of total analyzed apical dendrite length (20 μm from the cell body) extending from granule cells within the molecular layer of the dentate gyrus and pyramidal cells within the stratum radiatum of the CA1 (B) and CA3 (C) regions of the dorsal hippocampus. Each dot represents a mouse. Two-way ANOVA F(2,33)interaction=15.52, F(1,33)treatment=10.36, F(2,33)condition=6.814, Bonferroni [file 12974_2020_1922_MOESM1_ESM.zip › Figure S5.PNG]

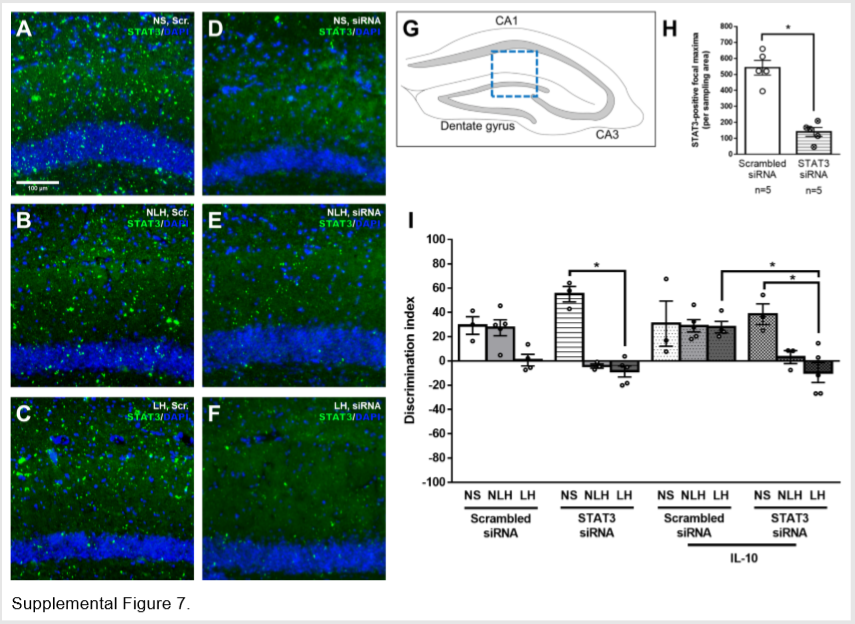

Supplement: Supplementary file 2 — Additional file 2: Supplemental Table 1: p-values for each figure. [file 12974_2020_1922_MOESM2_ESM.png]
